# Dinitrogen fixation in aphotic oxygenated marine environments

**DOI:** 10.3389/fmicb.2013.00227

**Published:** 2013-08-27

**Authors:** Eyal Rahav, Edo Bar-Zeev, Sarah Ohayon, Hila Elifantz, Natalia Belkin, Barak Herut, Margaret R. Mulholland, Ilana Berman-Frank

**Affiliations:** ^1^Mina and Everard Goodman Faculty of Life Sciences, Bar-Ilan UniversityRamat Gan, Israel; ^2^Israel Oceanographic and Limnological Research, National Institute of OceanographyHaifa, Israel; ^3^Department of Ocean, Earth and Atmospheric Sciences, Old Dominion UniversityNorfolk, VA, USA

**Keywords:** dinitrogen fixation, diazotrophs, aphotic layer, oligotrophic, heterotrophic diazotrophs

## Abstract

We measured N_2_ fixation rates from oceanic zones that have traditionally been ignored as sources of biological N_2_ fixation; the aphotic, fully oxygenated, nitrate (NO^−^_3_)-rich, waters of the oligotrophic Levantine Basin (LB) and the Gulf of Aqaba (GA). N_2_ fixation rates measured from pelagic aphotic waters to depths up to 720 m, during the mixed and stratified periods, ranged from 0.01 nmol N L^−1^ d^−1^ to 0.38 nmol N L^−1^ d^−1^. N_2_ fixation rates correlated significantly with bacterial productivity and heterotrophic diazotrophs were identified from aphotic as well as photic depths. Dissolved free amino acid amendments to whole water from the GA enhanced bacterial productivity by 2–3.5 fold and N_2_ fixation rates by ~2-fold in samples collected from aphotic depths while in amendments to water from photic depths bacterial productivity increased 2–6 fold while N_2_ fixation rates increased by a factor of 2 to 4 illustrating that both BP and heterotrophic N_2_ fixation were carbon limited. Experimental manipulations of aphotic waters from the LB demonstrated a significant positive correlation between transparent exopolymeric particle (TEP) concentrations and N_2_ fixation rates. This suggests that sinking organic material and high carbon (C): nitrogen (N) micro-environments (such as TEP-based aggregates or marine snow) could support high heterotrophic N_2_ fixation rates in oxygenated surface waters and in the aphotic zones. Indeed, our calculations show that aphotic N_2_ fixation accounted for 37 to 75% of the total daily integrated N_2_ fixation rates at both locations in the Mediterranean and Red Seas with rates equal or greater to those measured from the photic layers. Moreover, our results indicate that that while N_2_ fixation may be limited in the surface waters, aphotic, pelagic N_2_ fixation may contribute significantly to new N inputs in other oligotrophic basins, yet it is currently not included in regional or global N budgets.

## Introduction

In many of the world's oceans, the availability of dissolved inorganic nitrogen (N) controls primary production in surface waters (Falkowski, 1997; Karl et al., 2002). This is because the most abundant form of N in the oceans, dinitrogen (N_2_), is biologically unavailable to the majority of organisms that reside there. Subgroups of prokaryotic organisms (diazotrophs) fix N_2_ and convert it to ammonium via the nitrogenase enzyme complex. This biological process is an important source of “new” N to oceanic systems that can stimulate production and growth of microbial communities. Current models suggest that new N inputs from N_2_ fixation by cyanobacterial diazotrophs in the upper photic layers of the oceans is about 130–140 Tg N y^−1^ (Gruber, 2008; Eugster and Gruber, 2012).

Globally, on geologic timescales, N inputs via N_2_ fixation are thought to be balanced by N losses via denitrification (Falkowski, 1997; Deutsch et al., 2007). However, at present, rate measurements suggest that N losses from the ocean via denitrification (~245–322 Tg N y^−1^) far exceed N inputs via N_2_ fixation and terrestrial sources (Codispoti et al., 2001; Gruber and Galloway, 2008). The balance between N_2_ fixation and denitrification (including annamox) is considered to critically impact the oceanic inventory of bioavailable nitrogen and thus primary productivity (PP) in the oceans (Gruber and Galloway, 2008).

Current models generally restrict oceanic N_2_ fixation to the surface, sunlit, photic layer of the oceans where autotrophic cyanobacterial diazotrophs occur (Gruber, 2008). However, recent work suggests that prokaryotic N_2_ fixers are more widely distributed geographically than previously thought (Moisander et al., 2010; Mulholland et al., 2012) and diazotrophs have been identified from more diverse pelagic environments including aphotic pelagic waters in oxygen minimum zones (OMZ) (Hamersley et al., 2011; Jayakumar et al., 2012). While it is known that diazotrophs inhabit shallow benthic environments and microbial mats (Capone, 1983; Bebout et al., 1987; Herbert, 1999), sea grass communities (McGlathery, 2008), coral reefs (O'Neil and Capone, 2008), and even hydrothermal vent communities (Mehta and Baross, 2006), the small areal extent of these habitats is thought to limit their contribution to marine N_2_ fixation (Brooks et al., 1971; Carpenter and Capone, 2008).

For many years, investigations of marine N_2_ fixation also focused on oligotrophic oceanic regions because the presence of fixed N was considered inhibiting to active N_2_ fixation (Mulholland and Capone, 1999; Flores and Herrero, 2005; Ward et al., 2009). Oligotrophic regions harbor a variety of cyanobacterial diazotrophs including the filamentous cyanobacteria *Trichodesmium* spp., diatom-diazotroph associations (DDA's), and unicellular cyanobacteria such as *Croccosphaera* and group A cyanobacteria (UCYN-A) (Bar-Zeev et al., 2008; Carpenter and Capone, 2008; Zehr et al., 2008). However, even *Trichodesmium*, the most widely studied marine diazotroph, can fix N_2_ when ambient inorganic N concentrations are on ~5 μ M (Mulholland et al., 2001), conditions rarely reached in most oceanic photic realms. N_2_ fixation of marine diazotrophs has been observed in nutrient replete cultures (Mulholland et al., 2001) and systems with high dissolved inorganic N concentrations (Voss et al., 2004; Rees et al., 2009; Mulholland et al., 2012). Data compilation from euphotic, mesopelagic, and benthic studies shows that NO^−^_3_ and NH^+^_4_ concentrations, up to 30 and 200 μM respectively, can result in N_2_ fixation rates similar to those found in nitrate depleted surface waters (reviewed in Knapp, 2012). Moreover, different sensitivities to DIN concentrations, exposure time, and compensation mechanisms all suggest that dissolved N concentrations do not control diazotroph distribution and activities in many marine environments (Knapp, 2012).

In addition to photoautotrophic diazotrophs that have been previously considered the dominant N_2_ fixers in marine systems (Carpenter and Capone, 2008), heterotrophic diazotrophs from α, β, γ, δ, and ε-proteobacterial phylogenies, as well as methanogenic archaea contribute to marine N_2_ fixation including benthic and pelagic realms, mesotrophic and eutrophic environments (Chien and Zinder, 1996; Wu et al., 2003; Zehr et al., 2003; Halm et al., 2012). Heterotrophic diazotrophs have been identified from NO_3_ -rich deep, aphotic zones of the oceans (Riemann et al., 2010), including OMZ (Jayakumar et al., 2012) and from near hydrothermal vents (Mehta et al., 2005). In the subtropical Atlantic, cyanobacterial *nifH* genes dominated the photic layer (Langlois et al., 2005); while bacterial *nifH* genes dominated the deep aphotic zones (Riemann et al., 2010). In the ultraoligotrophic South Pacific Gyre (SPG) sequence analyses of *nifH* genes and their expressed transcripts revealed a prevalence of heterotrophic diazotrohic γ-proteobacteria (Cluster I—Zehr et al., 2003) and the unicellular photoheterotrophic Group A cyanobacteria (UCYN-A) down to 200 m- the deep chlorophyll maximum (Halm et al., 2012).

While the presence of diazotrophs in oceanic aphotic zones has been demonstrated, to our knowledge, only three published studies have actually measured rates of N_2_ fixation in aphotic environments; one study was conducted in the Eastern Tropical Pacific (Fernandez et al., 2011), another in the Southern California Bight (Hamersley et al., 2011), and the third in a hydrothermal vent community (Mehta and Baross, 2006). The first two studies were located in OMZ or hypoxic areas. In the Southern California Bight aphotic N_2_ fixation accounted for up to one-third of the total areal rates of N_2_ fixation (55 μmol N m^−2^ d^−1^ out of 150 μmol N m^−2^ d^−1^) (Hamersley et al., 2011). While in the nutrient-rich OMZ of the Eastern Tropical South Pacific (ETSP), N_2_ fixation rates from sub-surface oxygen deficient waters contributed about 5 times more N than N_2_ fixation from the oxic photic layer (Fernandez et al., 2011). Moreover, N_2_ fixation co-occurred spatially and temporally with denitrification in the ETSP (Fernandez et al., 2011), refuting the paradigm of spatial and/or temporal uncoupling between the two processes.

In oligotrophic environments such as the Levantine Basin (LB) (Eastern Mediterranean Sea, EMS) and the Gulf of Aqaba (northern Red Sea, GA), primary production in surface waters is limited at times by nitrogen (N), phosphorus (P), or co-limited by both (Paytan et al., 2009; Krom et al., 2010; Tanaka et al., 2011). Denitrification in subsurface waters is absent because waters are fully oxygenated (Kress and Herut, 2001; Al-Qutob et al., 2002). In the EMS, new nitrogen inputs via N_2_ fixation in the photic zone are generally very low, with rates ranging from 0.01 to 0.3 nmol N L^−1^ d^−1^. These rates contribute only 1–2% of new production in the LB (Yogev et al., 2011; Berman-Frank and Rahav, 2012; Rahav et al., 2013a). Although occasional small blooms of *Trichodesmium* and DDA's occur in the GA, N_2_ fixation rates are still relatively low ranging from ~0.05 to 2 nmol N L^−1^ d^−1^ (Post et al., 2002; Foster et al., 2009). In the photic layer of the EMS and the GA, diazotroph populations include cyanobacteria as well as many heterotrophic microbes (Man-Aharonovich et al., 2007; Foster et al., 2009; Yogev et al., 2011).

In this study we measured N_2_ fixation rates in the aphotic, oxygenated waters of the oligotrophic LB and the GA. Our results demonstrate significant N_2_ fixation rates equal or greater to those measured from the photic layers and a prevalence of heterotrophic diazotrophs. This initial data suggests the aphotic oxygenated zones can be considerable sources of new N supporting biological production in oligotrophic waters.

## Materials and methods

### Sampling locations

Seawater samples were collected from two representative pelagic stations that were sampled several times during the mixed and stratified periods; one in the GA in the Red Sea and one in the LB in the Eastern Mediterranean Sea (Table 1 and below). Water samples were collected using 10 L Niskin bottles mounted on a rosette equipped with a CTD (Seabird 19 Plus) and fluorometer (Turner designs, Cyclops7) to measure hydrographic properties of the water column, and an *in situ* PAR sensor (QSP-2300, Biospherical Instruments Inc) allowing distinction between the photic and aphotic zones.

**Table 1:** **Summary of the sampling dates, locations, and depths**.

**Region**	**Sampling location**		**Sampling duration**	**Bottom depth (m)**	**Photic depths sampled (m)**	**Aphotic depths sampled (m)**	**Depth of 0.1% light (m)**
Red Sea	Gulf of Aqaba (GA)	29° 55N′	Mar. 2010	720	0,20,50,100	150	105
		34° 29E′	Aug. 2010		0,50, 100	200,300,500	120
			July 2012		10,85	160, 300, 500, 720	100
Med. Sea	Levantine basin (LB)	32° 57N′	Dec. 2010	1100	0,20,50,100	300, 500	110
		34° 45E′	Aug. 2011		5	250	140
			Oct. 2011		5	300	130
			Feb. 2012		5	300	110
			Apr.2012		5	300	110
			Mar. 2013		5, 80	250	100

Additional details are available in the Material and methods section. Med. Sea, Mediterranean Sea.

Gulf of Aqaba (GA)—Water samples from the GA were collected aboard the *R/V Sam Rothberg* at a station located at the northern tip of the Red Sea (Station A 29° 55′N, 34° 28′E) during the winter when the water column was well-mixed (March 2010) and twice during the stratified summer period (September 2010 and July 2012).

Levantine Basin (LB)—Samples from the LB were collected during cruises aboard the *R/V Mediterranean Explorer* and the *R/V Shikmona*. A station located west of the Israeli coastline (Station OUT; 32° 57′N, 34° 45′E) was sampled during periods when the water column was well mixed (December 2010, February 2012, March 2013) and stratified (August 2011, October 2011, April 2012).

### Inorganic nutrients

Duplicate unfiltered water samples were collected in 15-mL acid-washed plastic scintillation vials and immediately frozen at −20°C until they were analyzed with a segmented flow Skalar SANplus System Instrument as detailed in Kress and Herut (2001). The precisions of nitrate+ nitrite (hereafter NO_3_), ortho-phosphate (PO_4_) and silicic acid (Si(OH)_4_) measurements were 0.02, 0.003 and 0.06 μM, respectively. The limits of detection were 0.075 μM, 0.008 μM and 0.03 μM for NO_3_, PO_4_and Si(OH)_4_, respectively.

### Dinitrogen (N_2_) fixation rates

Rates of N_2_ fixation were measured in field- collected samples (4.5 L) using the ^15^N_2_ bubble addition technique described by Montoya et al. (1996) and Mulholland et al. (2006) using highly enriched ^15^N_2_ gas (99%). The seawater was dispensed into 4.5 L Nalgene incubation bottles, 9 mL of ^15^N_2_ was added, and bottles were then placed in an incubator with continuously flowing seawater to maintain ambient surface-water temperatures. Triplicate bottles were used for each depth sampled. The incubator was shaded with neutral density screening to simulate ambient irradiance conditions at the depths from which samples were collected. For samples collected from the aphotic layer, bottles were incubated in the dark. Both light and dark incubations were 24–48 h in duration. While the bubble method may underestimate N_2_ fixation (Mohr et al., 2010; Großkopf et al., 2012; Wilson et al., 2012), particularly during short incubations (hours), our long incubation times should have minimized this problem (Mulholland et al., 2012; Rahav et al., 2013b). See also the discussion regarding underestimation of rates due to the bubble vs. enriched seawater method (Mohr et al., 2010). To terminate the incubations, water was filtered onto pre-combusted 25 mm GF/F filters (450°C for 4 h). Filters were dried and pelletized in tin disks and analyzed on a Europa 20/20 mass spectrometer equipped with an automated nitrogen and carbon analyzer preparation module. N_2_ fixation was calculated using a mixing model (Orcutt et al., 2001) as described by Mulholland et al. (2006) with N solubility factors according to Weiss (1970).

Depth-integrated N_2_ fixation rates were based on measurements made at 3 to 6 water column depths spanning both photic and aphotic layers. In months where N_2_ fixation measurements were made at only 2 depths, no such calculations were made (i.e., Not Available, N.A; Table 2). Depth-integrations included only the portion of the water column sampled (range was 150- 720 m in depth) and did not extend to the bottom except for one case in the GA during July 2012, when N_2_ fixation rates were measured also in bottom waters (Table 1). The percent contribution of the aphotic N_2_ fixation was calculated by difference after subtracting out the contribution of photic N_2_ fixation from the total integrated water column N_2_ fixation rate.

**Table 2:** **Measured physical, chemical, and biological properties of the photic and aphotic layers from the sampling stations in the Levantine basin and the Gulf of Aqaba averaged seasonally for when the water column was mixed (winter) and during thermal stratification (spring, summer, and fall)**.

**Location**	**Depth**	**Period**	**Temp. (°C)**	**Salinity**	**NO_2_+ NO_3_(μM)**	**PO_4_(μM)**	**BP (μg C L^−1^d^−1^)**	**PP (μg C L^−1^d^−1^)**	**N_2_fixation (nmol N L^−1^d^−1^)**	**Contribution of layer to total N_2_fixation (%)**
Gulf of Aqaba	Photic	Mixed	22–23	40.5–40.7	0.09–0.90	BLD–0.04	0.4–0.8	0.1–3.1	0.05–0.11	63
		Stratified	23–27	40.5–40.8	0.04–0.06	BLD–0.04	1.5–3.8	0.3–0.6	0.35–0.63	25
Levantine basin		Mixed	17–18	39.1–39.2	0.06–0.46	0.02–0.10	0.4–0.5	0.7–2.3	BLD–0.01	44
		Stratified	18–28	39.2–39.5	BLD–0.20	BLD–0.03	0.4–2.5	0.4–1.8	0.01–0.17	N.A
Gulf of Aqaba	Aphotic	Mixed	21–22	40.5–40.7	0.80–5.61	0.04–0.36	0.2–0.4	0–0.1	0.02–0.20	37
		Stratified	21–23	40.4–40.7	0.06–6.24	0.04–0.36	0.5–2.4	0	0.07–0.38	75
Levantine basin		Mixed	14–18	38.9–39.1	0.20–4.31	0.10–0.25	0.8–2.7	0.3–0.5	0.01–0.02	56
		Stratified	15–18	38.9–39.1	0.10–4.52	0.10–0.20	4.6–10.7	N.A	0.01–0.24	N.A

N.A, not available, BLD, below detection limit.

### Primary productivity

Photosynthetic carbon fixation rates were estimated using ^13^C-labeled bicarbonate (Mulholland and Bernhardt, 2005). Water samples were placed in triplicate clear 4.5 L polycarbonate Nalgene bottles and amended with 99% NaH^13^CO_3_ (Sigma) to obtain enrichments of about 1% of the ambient dissolved inorganic carbon pool. Bottles were incubated under the same conditions as for the ^15^N_2_ incubation bottles described above. Parallel dark bottles were also incubated for photic depths. As for ^15^N_2_, ^13^C incubations were terminated by immediately filtering the entire contents of incubation bottles onto pre-combusted 25 mm GF/F filters (450°C for 4 h). Filters were stored at −20°C and then dried and pelletized in tin disks prior to their analysis on the Europa 20/20 mass spectrometer. Rates of C uptake were calculated using a mixing model (as described above) and dark carbon fixation was subtracted from the light carbon fixation rates for photic incubations.

### Bacterial productivity

Bacterial production was estimated using the ^3^H-leucine (Amersham, Specific activity: 160 Ci mmol^−1^) incorporation method (Simon et al., 1990). Triplicate 1.7 ml samples were incubated for 4–8 h at *in situ* temperatures in the dark. Triplicate, trichloroacetic acid (TCA) killed samples (Sigma T9159) served as controls. One ml of high ^3^H affinity scintillation cocktail (ULTIMA-GOLD) was added to samples and they were counted using a TRI-CARB 2100 TR, PACKARD scintillation counter.

### Amino-acids enrichment experiment

We assumed that enriching the water with amino acids would promote conditions that were advantageous to heterotrophic bacteria in general and also to heterotrophic diazotrophs. We assumed that if heterotrophic diazotrophic bacteria were limited by energy (carbon), the addition of high-carbon containing amino acids would alleviate this limitation allowing these populations to fix N_2_. The idea was to distinguish general bacterial heterotrophic activity (bacterial production rates) from diazotrophic ones (N_2_ fixation rates). The response of the microbial community to additions of an amino acid mixture (Sigma A9906) was tested at 4 aphotic (160, 300, 500, 720 m) and two photic (10, 85) depths (Tables 1, 2) during July 2012 in the GA. Amino acids were added to 4.5 L polycarbonate Nalgene bottles filled with whole water from a specified depth at a final concentration of 500 nM C immediately prior to making ^15^N_2_ additions.

### Pyrosequencing

DNA was extracted from a representative aphotic depth in the LB stations. Fifteen L of seawater were filtered onto 0.2 μm Supor® filter (PALL) and stored in −80°C until analysis in the lab. DNA was extracted using a genomic DNA kit (Roche) and the extract was purified with a QIAGEN purification kit according to the manufacturer's instructions. Amplification of *nif*H genes from genomic DNA was performed as described elsewhere (Zehr and McReynolds, 1989). The *nifH* sequences retrieved from the pyrosequencing analyses [performed by Research and Testing Laboratories (MRDNA, Texas, USA)] were trimmed and checked for quality using QIIME (Caporaso et al., 2010). The cleaned sequences were compared to a *nifH* database (Zehr et al., 2003). In the aphotic layer of the LB only 20 sequences with an open reading frame were retrieved. These sequences were further confirmed using BLASTX (Altschul et al., 1990) with a cutoff of 95%. The *nifH*-like sequences were integrated into a *nifH* phylogenetic tree created with MEGA 4.0 software described in details by Tamura et al. (2007). *nifH* gene sequences were submitted to GeneBank and were assigned the accession numbers KC222033–KC222052.

### Determination of transparent exopolymer particles (TEP) concentration and size

Triplicate water samples (100 ml) were filtered onto a 0.4 μm polycarbonate filters and stained for 2 s with a 0.02% Alcian Blue solution. TEP concentrations (μ g GX equivalents L^−1^) were measured according to Passow and Alldredge (1995). A conversion factor of 0.51 was used to convert from micrograms of Gum Xanthan (GX) equivalents to micrograms of carbon (Engel and Passow, 2001).

For TEP size (area - μm^2^) measurements, samples (100 ml) were filtered gently (~100 mbar) onto 0.4 μm polycarbonate filters, stained for 10 s with freshly prepared Alcian Blue solution (0.02%), rinsed with 5 ml of double distilled water (DDW), placed onto microscope slides and kept at 4°C. The filters were analyzed under X40 magnification after 1–2 days and at least 90 pictures per slide were taken. The pictures were analyzed with image J software (http://rsbweb.nih.gov/ij/) to determine the size of TEP.

### TEP enrichment experiment

The association of aphotic diazotrophs with TEP was explored by adding 4 different quantities of pure GX (Sigma G1253) to LB seawater taken from 250 m (March 2013). Three replicate bottles were tested per treatment and per unamended controls containing seawater only. The bottles were incubated in the dark at ambient temperatures (15°C) for 24 h, after which they were sampled to measure TEP concentration and size and rates of N_2_ fixation and BP.

### Statistical analyses

Data were displayed as means with corresponding standard deviations for correlation and regression analyses between variables. Comparisons between unamended controls (no addition) and the amended microcosms (+Dissolved free amino-acids, DFAA) at the end of the experiment, a One-Way ANOVA followed by Tukey's multiple comparison *post hoc* using R software (www.r-project.org). *P* < 0.05 was considered statistically significant.

## Results

### Physical and chemical characteristics of the aphotic layer

**Figure 1:**
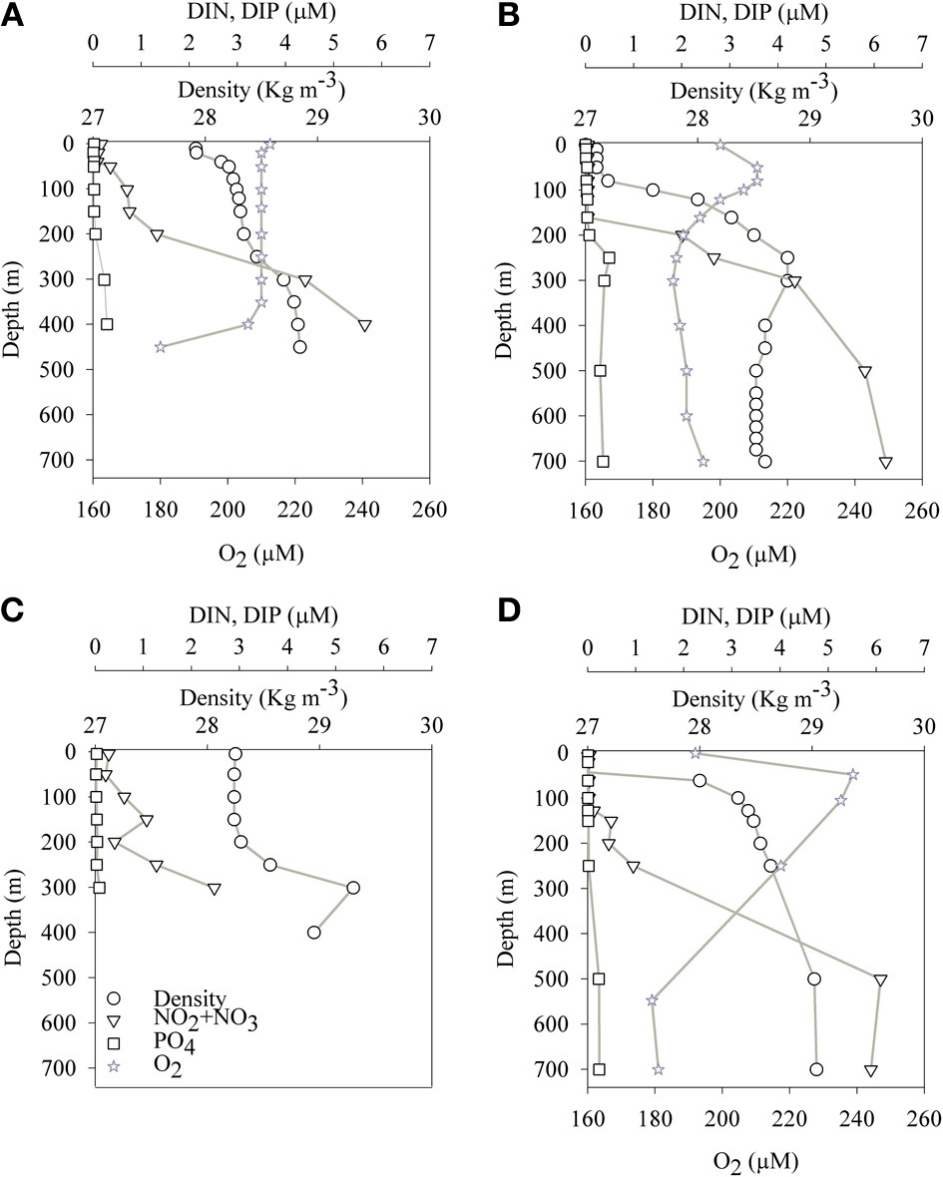
**Spatial (depth) and temporal changes of physical and chemical parameters at the sampling stations in the Gulf of Aqaba (A,B) and Levantine basin (C,D) during the mixed (A,C) and stratified (B,D) periods**.

The aphotic layer was determined at each station as the depth below which irradiance was <0.1% of surface irradiance. The depths of the photic layer varied between stations and seasons, ranging from 105 m to 140 m at both sampling locations (Table 1). Temperature and salinity in the aphotic layer of the GA station were 21.5 ± 0.5°C and 40.6 ± 0.1, respectively during both the mixed (winter) and stratified (summer) periods. In the photic layer of the GA, temperatures were 22 ± 0.5°C during winter and 25 ± 2°C during the stratified months (Figures 1A,B; Table 2). At the LB station, temperatures and salinities of the aphotic layer were significantly lower than those measured for the GA station, averaging 16.1 ± 1.1°C and 39.1 ± 0.1, respectively (Figures 1C,D; Table 2), while within the photic layer temperature and salinities averaged 23 ± 5°C and 39.3 ± 0.2, respectively, during the stratified period (Figures 1C,D; Table 2).

Average dissolved NO_3_ concentrations in the nitricline and sub-nitricline depths of the GA were 3.0 ± 2.1 μM with the nutricline ranging between 200–400 m during the winter mixing (*n* = 9, Figure 1A; Table 2). NO_3_ concentrations remained similar (3.0 ± 2.5 μM) during the stratified summer for the same depths although the nutricline deepened to 150–500 m (*n* = 13, Figure 1B; Table 2). PO_4_ (DIP) concentrations varied similarly in both periods, averaging 0.2 ± 0.2 μM (Figures 1A,B; Table 2). Surface NO_3_ and DIP concentrations were low and close to their detection limit during both periods (Figures 1A,B; Table 2).

Unlike the GA, no seasonal fluctuations in NO_3_ and DIP concentrations were recorded in the aphotic depths of the pelagic LB station (Figures 1C,D). At these depths lower NO_3_ and DIP concentrations were 2.2 ± 2.1 μM and 0.1 ± 0.5 μM, respectively (*n* = 7–9, Figures 1C,D; Table 2).

### N_2_ fixation rates

Photic N_2_ fixation rates compiled from both stations, at all depths and seasons yielded rates usually below 0.1 nmol N L^−1^ d^−1^ excluding rates measured in the GA during summer (0.35–0.63 nmol N L^−1^ d^−1^) (Table 2). Aphotic N_2_ fixation rates ranged from 0.01 to 0.38 nmol N L^−1^ d^−1^ at both locations sampled (Table 2). Aphotic rates obtained in the GA during the mixed period ranged from 0.02 to 0.2 nmol N L^−1^ d^−1^ and averaged 0.1 ± 0.1 nmol N L^−1^ d^−1^ while during the stratified period rates ranged from 0.07 to 0.38 nmol N L^−1^ d^−1^ and averaged 0.30 ± 0.10 nmol N L^−1^ d^−1^ (Table 2). At the LB station, aphotic N_2_ fixation was uniformly low during the mixed period; 0.01 ± 0.01 nmol N L^−1^ d^−1^ and increased ~20-fold during the stratified period to 0.24 ± 0.09 nmol N L^−1^ d^−1^ (Table 2).

To understand the significance of aphotic N_2_ fixation relative to the total areal rates of N_2_ fixation, we calculated that during our sampling times, N_2_ fixation from aphotic depths sampled accounted for 37 to 75% of total areal N_2_ fixation at the GA station and ~50% of the total areal N_2_ fixation at the LB station during the mixed period (Table 2).

### Phytoplankton and bacterial productivity rates

PP rates in the GA averaged 3.0 ± 0.1 μg C L^−1^ d^−1^ and 0.5 ± 0.1 μg C L^−1^ d^−1^ for the mixed and stratified periods, respectively (*n* = 3, PAR at surface of 1000–1500 μmol quanta m^−2^ s^−1^). PP at the LB station was generally lower; averaging 2.1 ± 0.2 μg C L^−1^ d^−1^ and 1.8 μg C L^−1^ d^−1^ for the mixed and stratified periods, respectively (*n* = 3, PAR at surface of 1500-1800 μmol quanta m^−2^ s^−1^) (Table 2). Bicarbonate uptake rates were usually below detection limits within the aphotic zone (below 105–140 m, depending on sampling location) excluding a wintertime measurement in the LB station (200 m) when C uptake averaged 0.4 ± 0.1 μg C L^−1^ d^−1^ (Table 2).

**Figure 2:**
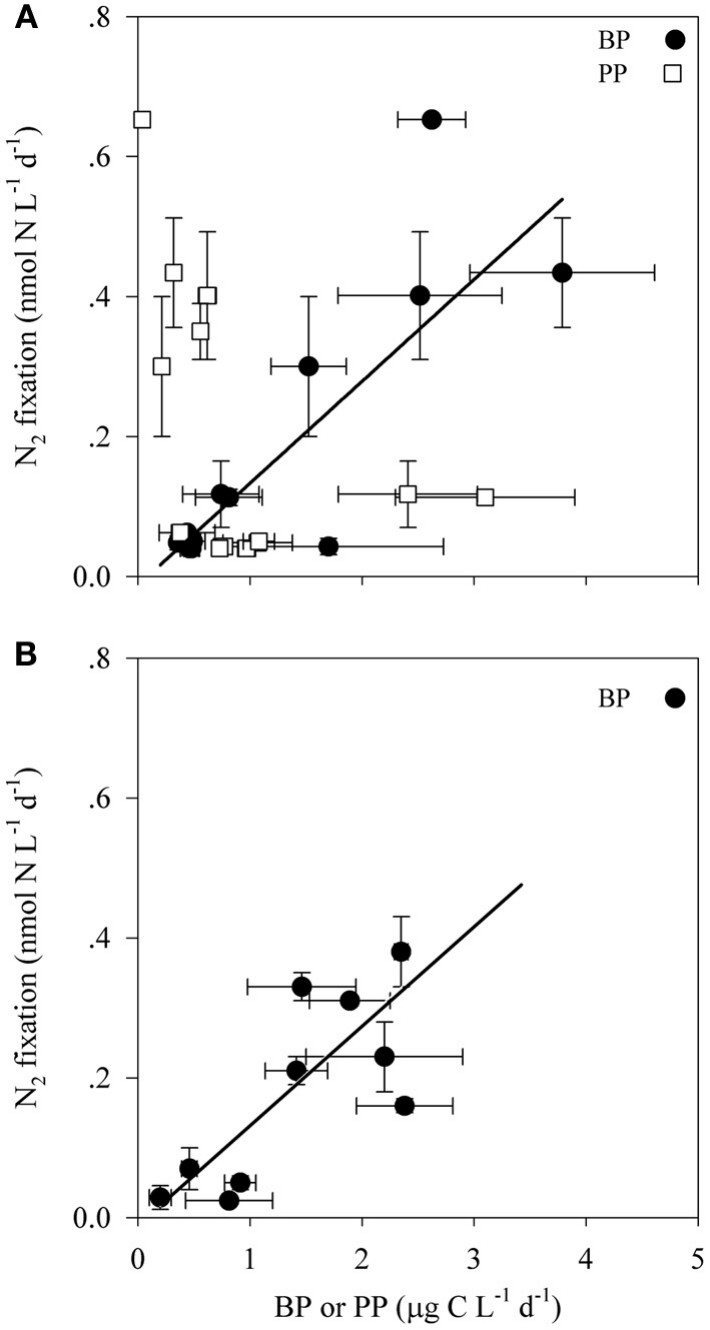
**The relationship between N_2_ fixation and primary and bacterial productivity within the photic **(A)** and aphotic **(B)** layers of the LB and GA stations.** Values are averages and error bars are standard deviations of three independent replicates performed for each control and treatment incubation.

Bacterial productivity (BP) rates in the photic waters from both locations ranged from 0.4 to 3.8 μg C L^−1^ d^−1^, while in the aphotic zone BP rates were 0.2 to 10.7 μg C L^−1^ d^−1^ (Table 2). Rates of N_2_ fixation from both the photic and aphotic layers were compared to BP and PP (Figure 2). BP correlated significantly and positively with N_2_ fixation rates within the photic zone of both basins (*R*^2^ = 0.70, *P* = 0.001, *n* = 12), while there was no apparent correlation between N_2_ fixation rates and PP (Figure 2A). Moreover, BP and N_2_ fixation were also positively correlated within the aphotic depths (*R*^2^ = 0.58, *P* = 0.003, *n* = 10) (Figure 2B).

To further understand what limits N_2_ fixation by heterotrophic diazotrophs in oligotrophic and aphotic systems we added dissolved free amino acids (DFAA) to microcosm experiments conducted in the oligotrophic stratified period during July 2012 in the GA. In water samples collected from the aphotic layer, DFAA additions enhanced BP rates by a factor of 2–3.5 and N_2_ fixation rates increased by a factor of 1.5–2 (ANOVA, *P* < 0.05) (Table 3). Within the photic layer, the response of bacteria to DFAA additions was greater than that observed in the aphotic zone; BP increased 2- to 6-fold and N_2_ fixation rates increased by a factor of 2 to 4 (ANOVA, *P* < 0.05) (Table 3).

**Table 3:** **The effect of dissolved free amino acid (DFAA) enrichments (+ DFAA) on bacterial productivity (BP) and N_2_ fixation during the stratified summer period (GA, July 2012) within different depths of the photic and aphotic layers**.

	**Sampling depth (m)**	**BP (μg C L^−1^d^−1^)**		**N_2_fixation (nmol N L^−1^d^−1^)**	
		**Control**	**+ DFAA**	**Control**	**+ DFAA**
Photic	10	0.30 ± 0.40	2.40 ± 0.38	0.35 ± 0.09	0.76 ± 0.10
	85	1.15 ± 0.33	4.17 ± 0.68	0.30 ± 0.11	1.12 ± 0.27
Aphotic	160	2.34 ± 0.69	4.68 ± 0.14	0.38 ± 0.05	0.70 ± 0.20
	300	2.20 ± 0.70	5.32 ± 0.20	0.23 ± 0.05	0.34 ± 0.02
	500	0.46 ± 0.07	1.57 ± 0.19	0.07 ± 0.03	0.13 ± 0.06
	720	2.38 ± 0.43	4.8 6± 0.47	0.16 ± 0.10	0.31 ± 0.06

Measurements are based on 3-4 replicates for each sampled depth.

### TEP as source of carbon or microhabitat

**Figure 3:**
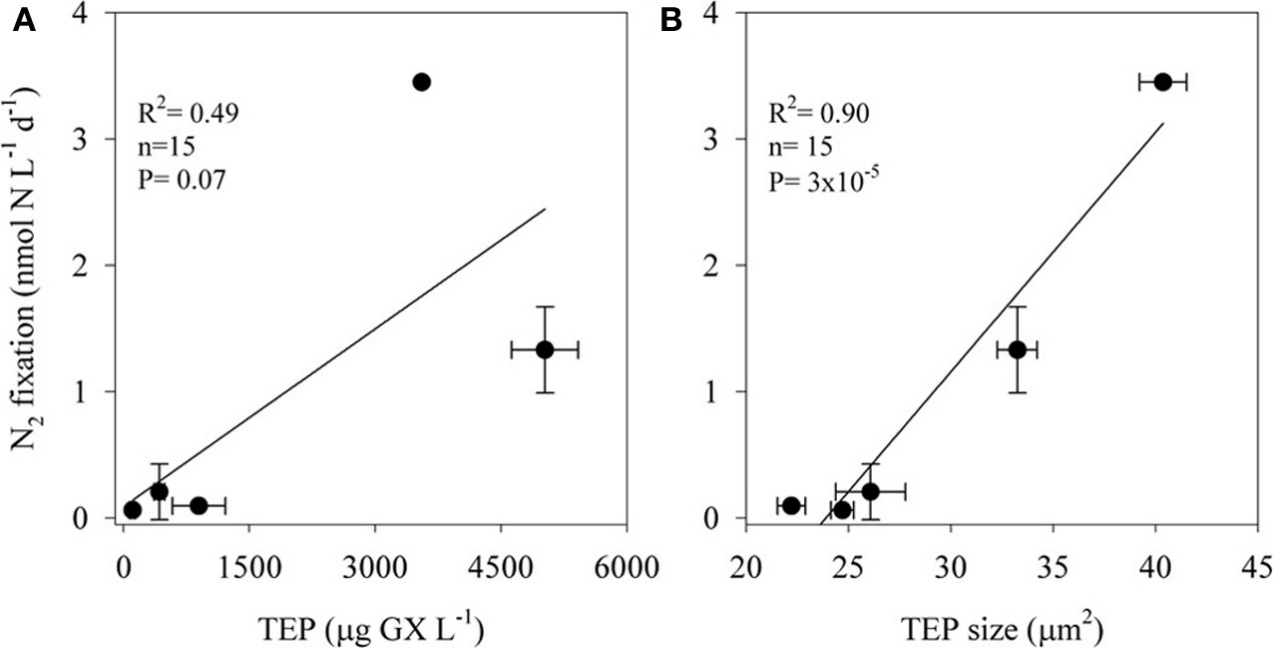
**The relationship between N_2_ fixation rates of aphotic diazotrophs and transparent exopolymer particles (TEP) concentrations **(A)** and size **(B)** in experimental manipulations of LB waters from 250 m depth to which we added 4 concentrations of pure gum xanthan.** Values are averages and error bars are standard deviations of three independent replicates performed for each control and treatment incubation.

TEP concentrations ranged from 100 to 5000 μg GX L^−1^ and varied in size; the two-dimensional TEP size ranged from 24 to 40 μm^2^ with the largest particles measured at TEP concentrations of ~3500 μg GX L^−1^ (Figure 3). BP increased almost 7-fold, from ~0.6 μg C L^−1^ d^−1^ in the unamended control bottles to ~4 μg C L^−1^ d^−1^ in incubations amended with the highest TEP concentrations (5000 μg GX L^−1^). N_2_ fixation was also stimulated (~57-fold) by TEP additions increasing from 0.06 nmol N L^−1^ d^−1^ in the controls to 3.46 nmol N L^−1^ d^−1^ in the bottles amended with ~3500 μg GX L^−1^. N_2_ fixation rates were positively correlated with both TEP concentration (*R*^2^ = 0.49, *P* = 0.07, *n* = 15) and TEP size (*R*^2^ = 0.90, *P* = 3 × 10^−5^, *n* = 15) (Figure 3).

### Molecular diversity of *nifH*

**Figure 4:**
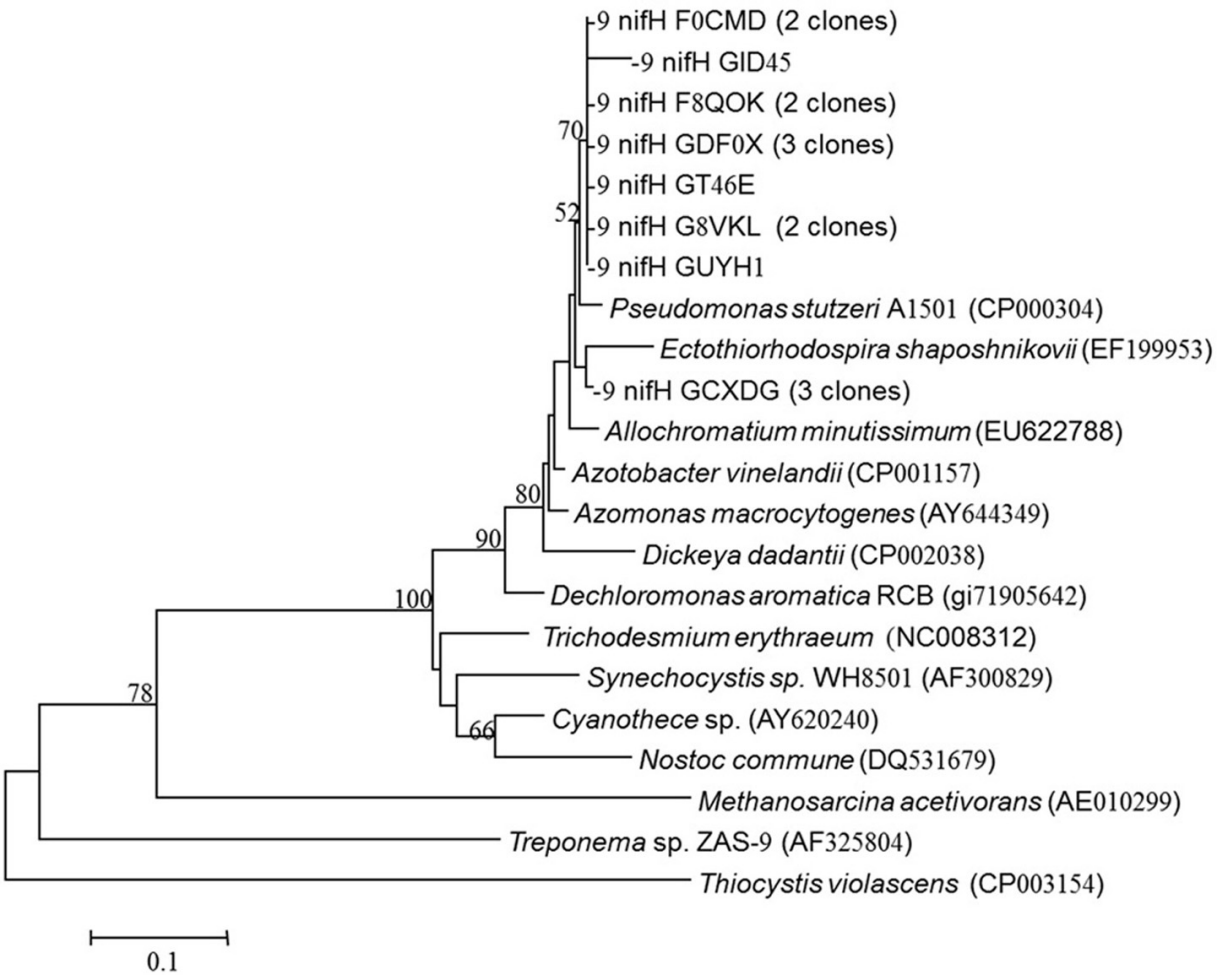
***nifH* gene tree of clones retrieved from the aphotic layer in the Levantine Basin.** Numbers in parentheses represent the number of clones in the library that were identical to the clones in the phylogenetic tree. Bootstrap values above 50% are indicated above the nodes.

*NifH*-like genes were retrieved from the aphotic layer of the LB and were compared with *nifH* genes from known bacterial isolates. Twenty sequences were retrieved and all clustered together with heterotrophic bacteria and were most similar to *Pseudomonas stutzeri* (Figure 4). All of our sequences were separated from the autotrophic clusters.

## Discussion

Most marine N_2_ fixation studies neglect the potential contribution of biological N_2_ fixation in deep, aphotic, NO_3_-rich waters that comprise the majority of the world's oceans (Karl et al., 2002; Zehr and Ward, 2002). This is likely because phototrophic and cyanobacterial diazotrophs have an ecological advantage in surface waters where productivity is often limited by the availability of fixed nitrogen yet there is sufficient energy (irradiance) to drive both photosynthetic C uptake and the costly process of N_2_ fixation (Howarth et al., 1988; Postgate, 1998; Karl et al., 2002). Currently these assumptions are being modified as significant N_2_ fixation rates are reported from marine areas with high DIN concentrations (review in Knapp, 2012). Here we provide evidence that aphotic, nitrogen-rich waters also harbor active diazotrophs that contribute substantial new N to the oceanic N inventory, and that N_2_ fixation by these bacterial diazotrophs may be limited by the supply of organic C.

In this study we have documented for the first time significant N_2_ fixation rates from the aphotic, oxidized waters (Figure 1) of the oligotrophic LB and the GA. The measured N_2_ fixation rates in the aphotic waters of both LB and GA ranged from 0.01 nmol N L^−1^ d^−1^ to 0.38 nmol N L^−1^ d^−1^. These rates were similar to the N_2_ fixation rates reported from aphotic depths in the hypoxic Southern California Bight (Hamersley et al., 2011), yet were ~10–100 fold lower than those measured in the Peruvian upwelling OMZ (Fernandez et al., 2011).

The significance of our measured rates was realized upon integration of water column N_2_ fixation from surface to the deepest depth sampled (Table 1). Thus, the contribution of aphotic N_2_ fixation accounted for ~50% from the total N_2_ fixation in the LB station, where winter mixing is shallow and the aphotic water hardly mixes with the photic layer, and 37 to 75% within the GA station (Table 2).

The substantial role of aphotic N_2_ fixation in the LB and the GA may be especially important to basin-wide productivity given the low rates of N_2_ fixation in the photic layers of both these nutrient-poor environments (Foster et al., 2009; Berman-Frank and Rahav, 2012). Within the photic layer, N_2_ fixation was undetectable in a third of the measurements made in the Eastern Mediterranean Sea during several studies (*n* > 250) spanning seasons and years (Ibello et al., 2010; Yogev et al., 2011). In contrast, measureable rates of N_2_ fixation were observed in >90% of samples collected from the aphotic zone (*n* = 48), regardless of season, thermal stability or depth (Table 2), further emphasizing the importance of N_2_ fixation in these realms and the requirement for subsequent comprehensive investigations that could constrain modeling efforts.

Our measured aphotic N_2_ fixation rates may actually represent an underestimate of this process as: (1) our sampling did not cover the full depth of the water column—thus integration may yield higher contribution of aphotic to the total N_2_ fixation (see methods); (2) we used the common gas bubble enrichment method (Montoya et al., 1996 and see Materials and Methods) which may underestimate N_2_ fixation rates by a factor of 2–6 compared to the enriched seawater method (Großkopf et al., 2012; Wilson et al., 2012). Early field comparisons from the LB photic waters between the two methods showed a 2–3 fold underestimation by the gas bubble technique (Rahav et al., 2013b). Assuming our aphotic rates were underestimated by a similar ratio of ~50%, our measured aphotic N_2_ fixation would increase accordingly so that maximal aphotic rates would be ~0.8 nmol N L^−1^ d^−1^, and with DFAA addition up to 1.4 nmol N L^−1^ d^−1^. Yet, as the estimated increase by 50% would also be accounted for in photic rates of N_2_ fixation, the % contribution of aphotic to total N_2_ fixation would remain the same.

Energetic requirements for diazotrophy are high (Postgate, 1998), thus we initially assumed that phototrophic (mainly cyanobacterial) diazotrophs would dominate the photic zone, while at aphotic depths, N_2_ fixation rates would be extremely low and accomplished by heterotrophic bacterial populations that would be prone to carbon limitation due to the low overlying productivity. However, we found that in the LB and GA stations heterotrophic diazotrophs were the dominant N_2_ fixers in both photic and aphotic zones. This was confirmed by examining the relationship between N_2_ fixation and BP and PP (Figure 2), molecular analysis of *nifH* genes (Figure 4), and the stimulation of N_2_ fixation after amino acid amendments (Table 3). In the stratified photic zone, a significant correlation was found between BP and N_2_ fixation (*R*^2^ = 0.70, *P* = 0.001, *n* = 10) and both were stimulated by the addition of DFAA (Table 3). In contrast, no correlation was found between N_2_ fixation and PP (Figure 2A). Although ample solar irradiance was available, heterotrophic bacterial diazotrophs appear to dominate in this system relying on dissolved and particulate organic carbon (DOC and POC, respectively) as energy sources (Goldthwait et al., 2005; Azam and Malfatti, 2007; Eichinger et al., 2009).

Within the aphotic layer, N_2_ fixation was positively correlated with BP (*R*^2^ = 0.58, *P* = 0.003, *n* = 10), (Figure 2). Moreover, a 2–3 fold enhancement of N_2_ fixation rates and enhanced BP were observed following DFAA amendments (Table 3). DFAA are labile dissolved organic carbon (DOC) and nitrogen (DON) sources for microbes (Middelburg and Nieuwenhuize, 2001) that can be directly utilized by bacterial heterotrophs (Davis et al., 2009). Our data thus suggests that the addition of C and N sources stimulated both diazotrophic and non-diazotrophic heterotrophic populations. Moreover, the increase in N source concomitantly with enhanced N_2_ fixation rates demonstrate that the higher concentration of dissolved N sources (either DON or bacterially catabolized into DIN) were not sufficient to inhibit diazotrophy. These results highlight the significant role of heterotrophic bacteria as important components of the photic and aphotic diazotrophic community and suggest that the availability of organic matter may limit bacterial diazotrophy in these oligotrophic regimes.

Previous molecular characterization of diazotrophs that were actively expressing *nifH* genes from the photic zones of the LB and GA demonstrated a diverse array of both heterotrophic bacteria and cyanobacterial phylotypes from Clusters I, II, and III (Foster et al., 2009; Yogev et al., 2011). In this study, phylogenetic analyses of *nifH* from the aphotic depths indicated that diazotrophs present were within Clusters I and III, phylotypes predominantly comprised of heterotrophic bacteria, including α and γ proteobacteria (Chien and Zinder, 1996) (Figure 4). The *nifH* phylogenetic diversity obtained from the aphotic zone during this study was similar to that recovered from the Southern California Bight (Hamersley et al., 2011) and anaerobic bacteria from the Sargasso Sea (Hewson and Fuhrman, 2007) (Figure 4).

Our results suggest that in the aphotic zones of both LB and GA heterotrophic bacterial N_2_ fixation is a considerable source of new N. Yet these environments have not heretofore been considered as major sources of new oceanic N inputs, likely because they are dark, oxygenated, and NO_3_-rich (Figure 1). Heterotrophic diazotrophs in aphotic areas may be carbon (energy) limited, especially in oligotrophic systems where export of organic particulate matter to depth is low. This is illustrated by the uncoupling between BP and PP observed in our study sites. In this study we demonstrated that N_2_ fixation and BP rates were enhanced when DFAA were added (Table 3). In addition to labile organic compounds such as DFAA, the mesopelagic aphotic layer is rich in organic carbon in the form of planktonic microgelatinous marine snow (Azam, 1998; Azam and Malfatti, 2007). This includes protobiofilms that are transparent exopolymer particles (TEP), heavily colonized by bacteria (Bar-Zeev et al., 2011, 2012). In oligotrophic systems these protobiofilm shuttles contribute significantly to the organic carbon pool especially at depths where other C sources are scarce (Azam and Malfatti, 2007; Ward et al., 2009; Bar-Zeev et al., 2012). While these particles are rich in C, the high C:N ratios of TEP (Engel and Passow, 2001; Passow, 2002) make them poor N sources and would seem to require the use of some N supplement to foster microbial growth. Heterotrophic N_2_ fixation could provide an excellent source of N to fuel microbial growth on, within, and near these particles. Indeed, our data demonstrate a positive relationship between TEP concentration (*R*^2^ = 0.49, *P* = 0.07, *n* = 15), TEP size (*R*^2^ = 0.90, *P* = 3 × 10^−5^, *n* = 15), and N_2_ fixation (Figure 3). The ability to fix N_2_ could provide diazotrophic heterotrophs with a competitive advantage over non-diazotrophic heterotrophic bacteria in carbon-rich and nitrogen-poor micro-environments such as these. Moreover, in aggregates such as marine snow, TEP, and other marine microgels, bacterial colonization causes high metabolic consumption of oxygen and decreasing oxygen gradients from the surface to the anaerobic center (Paerl and Prufert, 1987). Reduced oxygen concentrations would theoretically benefit diazotrophic bacteria because nitrogenase is an oxygen-sensitive protein (Postgate, 1998). We speculate these anaerobic, high C, low N microenvironments could promote the competitive success and activity of heterotrophic diazotrophs at depth.

Our novel data thus provides a conservative estimate of an additional 37 to 75% source of N via aphotic N_2_ fixation in the oligotrophic Levantine basin and the GA. Our measurements substantiate early indications (Paerl and Prufert, 1987) and recent phylogenetic characterizations (Riemann et al., 2010) highlighting a pivotal ecological role for diazotrophs in deep, oxygenated, NO_3_-rich waters. Because these areas comprise the bulk of the world's oceans they could represent a hitherto unaccounted for source of new N that may contribute to global productivity and could reduce the existing discrepancies [along with other newly described contributions of N_2_ fixation (reviewed in Knapp, 2012)] between sources and losses of N found in global marine N budgets.

### Conflict of interest statement

The authors declare that the research was conducted in the absence of any commercial or financial relationships that could be construed as a potential conflict of interest.

## References

[B1] Al-Qutob M. Hase C. Tilzer M. M. Lazar B. (2002). Phytoplankton drives nitrite dynamics in the Gulf of Aqaba, Red Sea. Mar. Ecol. Prog. Ser. 239, 233–239 10.3354/meps239233

[B2] Altschul S. F. Gish W. Miller W. Myers E. W. Lipman D. J. (1990). Basic local alignment search tool. J. Mol. Biol. 215, 403–410 2231712 10.1016/S0022-2836(05)80360-2

[B3] Azam F. (1998). Microbial control of oceanic carbon flux: the plot thickens. Science 280, 694–696 10.1126/science.280.5364.694

[B4] Azam F. Malfatti F. (2007). Microbial structuring of marine ecosystems. Nat. Rev. Microbiol. 5, 782–791 10.1038/nrmicro1747 17853906

[B5] Bar-Zeev E. Berman T. Rahav E. Dishon G. Herut B. Berman-Frank I. (2011). Transparent exopolymer particle (TEP) dynamics in the Eastern Mediterranean Sea. Mar. Ecol. Prog. Ser. 431, 107–118 10.3354/meps09110

[B6] Bar-Zeev E. Berman-Frank I. Girshevitz O. Berman T. (2012). Revised paradigm of aquatic biofilm formation facilitated by microgel transparent exopolymer particles. Proc. Natl. Acad. Sci. U.S.A. 109, 9119–9124 10.1073/pnas.1203708109 22615362 PMC3384133

[B7] Bar-Zeev E. Yogev T. Man-Aharonovich D. Kress N. Herut B. Béjà O. (2008). Seasonal dynamics of the endosymbiotic, nitrogen-fixing cyanobacterium *Richelia intracellularis* in the Eastern Mediterranean Sea. ISME J. 2, 911–923 10.1038/ismej.2008.56 18580972

[B8] Bebout B. M. Paerl H. W. Crocker K. M. Prufert L. E. (1987). Diel interactions of oxygenic photosynthesis and N_2_ fixation (acetylene-reduction) in a marine microbial mat community. Appl. Environ. Microbiol. 53, 2353–2362 16347456 10.1128/aem.53.10.2353-2362.1987 PMC204112

[B9] Berman-Frank I. Rahav E. (2012). “Nitrogen fixation as a source for new production in the Mediterranean Sea: a review,” in Life in the Mediterranean Sea: A Look at Habitat Changes, ed Stambler N. E. (New York, NY: Nova Science Publishers), 199–226

[B10] Brooks R. H. Brezonik P. L. Putnam H. D. Keirn M. A. (1971). Nitrogen fixation in an estuarine environment: the Waccasassa on Florida Gulf coast. Limnol. Oceanogr. 16, 701–710 10.4319/lo.1971.16.5.0701

[B11] Capone D. G. (1983). N_2_ fixation in seagrass communities. Mar. Technol. Soc. J. 17, 32–37

[B12] Caporaso J. G. Kuczynski J. Stombaugh J. Bittinger K. Bushman F. D. Costello E. K. (2010). QIIME allows analysis of high-throughput community sequencing data. Nat. Methods 7, 335–336 10.1038/nmeth.f.303 20383131 PMC3156573

[B13] Carpenter E. J. Capone D. (2008). “Nitrogen fixation in the marine environment,” in Nitrogen Fixation in the Marine Environment, eds Capone D. Bronk A. D. Mulholland R. M. Carpenter E. J. (San Diego, CA: Elsevier Academic Press), 141–198

[B14] Chien Y. T. Zinder S. H. (1996). Cloning, functional organization, transcript studies, and phylogenetic analysis of the complete nitrogenase structural genes (nifHDK2) and associated genes in the archaeon *Methanosarcina barkeri 227*. J. Bacteriol. 178, 143–148 8550408 10.1128/jb.178.1.143-148.1996 PMC177631

[B15] Codispoti L. A. Brandes J. A. Christensen J. P. Devol A. H. Naqvi S. W. A. Paerl H. W. (2001). The oceanic fixed nitrogen and nitrous oxide budgets: moving targets as we enter the anthropocene. Sci. Mar. 65, 85–105

[B16] Davis J. Kaiser K. Benner R. (2009). Amino acid and amino sugar yields and compositions as indicators of dissolved organic matter diagenesis. Org. Geochem. 40, 343–352 10.1016/j.orggeochem.2008.12.003

[B17] Deutsch C. Sarmiento J. L. Sigman D. M. Gruber N. Dunne J. P. (2007). Spatial coupling of nitrogen inputs and losses in the ocean. Nature 445, 163–167 10.1038/nature05392 17215838

[B18] Eichinger M. Kooijman S. A. L. M. Sempere R. Lefevre D. Gregori G. Charriere B. (2009). Consumption and release of dissolved organic carbon by marine bacteria in a pulsed-substrate environment: from experiments to modeling. Aquat. Microb. Ecol. 56, 41–54 10.3354/ame01312

[B19] Engel A. Passow U. (2001). Carbon and nitrogen content of transparent exopolymer particles (TEP) in relation to their Alcian Blue adsorption. Mar. Ecol. Prog. Ser. 219, 1–10 10.3354/meps219001

[B20] Eugster O. Gruber N. (2012). A probabilistic estimate of global marine N-fixation and denitrification. Global Biogeochem. Cycles 26 10.1029/2012GB004300

[B21] Falkowski P. G. (1997). Evolution of the nitrogen cycle and its influence on the biological sequestration of CO_2_ in the ocean, Nature 387, 272–275 10.1038/387272a0

[B22] Fernandez C. Farías L. Ulloa O. (2011). Nitrogen fixation in denitrified marine waters. PLoS ONE 6:e20539. 10.1371/journal.pone.0020539 10.1371/journal.pone.0020539 21687726 PMC3110191

[B23] Flores E. Herrero A. (2005). Nitrogen assimilation and nitrogen control in cyanobacteria. Biochem. Soc. Trans. 33, 164–167 10.1042/BST0330164 15667295

[B24] Foster R. A. Paytan A. Zehr J. P. (2009). Seasonality of N_2_ fixation and *nifH* gene diversity in the Gulf of Aqaba (Red Sea). Limnol. Oceanogr. 54, 219–233 10.4319/lo.2009.54.1.0219

[B25] Goldthwait S. A. Carlson C. A. Henderson G. K. Alldredge A. L. (2005). Effects of physical fragmentation on remineralization of marine snow. Mar. Ecol. Prog. Ser. 305, 59–65 10.3354/meps305059

[B26] Großkopf T. Mohr W. Baustian T. Schunck H. Gill D. Kuypers M. M. M. (2012). Doubling of marine dinitrogen-fixation rates based on direct measurements. Nature 488, 361–364 10.1038/nature11338 22878720

[B27] Gruber N. (2008). “The marine nitrogen cycle: overview of distributions and processes,” in Nitrogen in the Marine Environment, 2nd Edn., eds Capone D. Bronk D. Mulholland M. Carpenter E. (Amsterdam: Elsevier), 1–50

[B28] Gruber N. Galloway J. N. (2008). An Earth-system perspective of the global nitrogen cycle. Nature 451, 293–296 10.1038/nature06592 18202647

[B29] Halm H. Lam P. Ferdelman T. G. Lavik G. Dittmar T. Laroche J. (2012). Heterotrophic organisms dominate nitrogen fixation in the South Pacific Gyre. ISME J. 6, 1238–1249 10.1038/ismej.2011.182 22170429 PMC3358028

[B30] Hamersley M. R. Turk K. A. Leinweber A. Gruber N. Zehr J. P. Gunderson T. (2011). Nitrogen fixation within the water column associated with two hypoxic basins in the Southern California Bight. Aquat. Microb. Ecol. 63, 193–205 10.3354/ame01494

[B31] Herbert R. A. (1999). Nitrogen cycling in coastal marine ecosystems. FEMS Microb. Rev. 23, 563–590 10.1111/j.1574-6976.1999.tb00414.x 10525167

[B32] Hewson I. Fuhrman J. A. (2007). Covariation of viral parameters with bacterial assemblage richness and diversity in the water column and sediments. Deep Sea Res. I. 54, 811–830 10.1016/j.dsr.2007.02.003

[B33] Howarth R. W. Marino R. Lane J. Cole J. J. (1988). Nitrogen-fixation in fresh-water, estuarine, and marine ecosystems.1. rates and importance. Limnol. Oceanogr. 33, 669–687 10.4319/lo.1988.33.4_part_2.0669

[B33a] Ibello V. Cantoni C. Cozzi S. Civitarese G. (2010). First basin-wide experimental results on N2 fixation in the open Mediterranean Sea. Geophys. Res. Lett. 37 10.1029/2009gl041635

[B34] Jayakumar A. Al-Rshaidat M. M. D. Ward B. B. Mulholland M. R. (2012). Diversity, distribution, and expression of diazotroph *nifH* genes in oxygen-deficient waters of the Arabian Sea. FEMS Microbiol. Ecol. 82, 597–606 10.1111/j.1574-6941.2012.01430.x 22697171

[B35] Karl D. Michaels A. Bergman B. Capone D. Carpenter E. Letelier R. (2002). Dinitrogen fixation in the world's oceans. Biogeochemistry 57, 47–98 10.1023/A:1015798105851

[B36] Knapp A. N. (2012). The sensitivity of marine N_2_ fixation to dissolved inorganic nitrogen. Front. Microbiol. 3:374 10.3389/fmicb.2012.00374 23091472 PMC3476826

[B37] Kress N. Herut B. (2001). Spatial and seasonal evolution of dissolved oxygen and nutrients in the Southern Levantine Basin (Eastern Mediterranean Sea): chemical characterization of the water masses and inferences on the N: P ratios. Deep Sea Res. I. 48, 2347–2372 10.1016/S0967-0637(01)00022-X

[B38] Krom M. D. Emeis K. C. Van Cappellen P. (2010). Why is the Eastern Mediterranean phosphorus limited. Prog. Oceanogr. 85, 236–244 10.1016/j.pocean.2010.03.003

[B39] Langlois R. J. Laroche J. Raab P. A. (2005). Diazotrophic diversity and distribution in the tropical and subtropical Atlantic Ocean. Appl. Environ. Microbiol. 71, 7910–7919 10.1128/AEM.71.12.7910-7919.2005 16332767 PMC1317331

[B40] Man-Aharonovich D. Kress N. Bar-Zeev E. Berman-Frank I. Beja O. (2007). Molecular ecology of *nifH* genes and transcripts in the Eastern Mediterranean Sea. Environ. Microbiol. 9, 2354–2363 10.1111/j.1462-2920.2007.01353.x 17686031

[B41] McGlathery K. J. (2008). “The marine nitrogen cycle: overview of distributions and processes,” in Nitrogen in the Marine Environment, 2nd Edn., eds Capone D. Bronk D. Mulholland M. Carpenter E. (Amsterdam: Elsevier), 1037–1071

[B42] Mehta M. P. Baross J. A. (2006). Nitrogen fixation at 92 degrees C by a hydrothermal vent archaeon. Science 314, 1783–1786 10.1126/science.1134772 17170307

[B43] Mehta M. P. Huber J. A. Baross J. A. (2005). Incidence of novel and potentially archaeal nitrogenase genes in the deep Northeast Pacific Ocean. Environ. Microbiol. 7, 1525–1534 10.1111/j.1462-2920.2005.00836.x 16156726

[B44] Middelburg J. J. Nieuwenhuize J. (2001). Nitrogen isotope tracing of dissolved inorganic nitrogen behavior in tidal estuaries. Estuar. Coast. Shelf S. 53, 385–391 10.1006/ecss.2001.0805

[B45] Mohr W. Grosskopf T. Wallace D. W. R. Laroche J. (2010). Methodological underestimation of oceanic nitrogen fixation rates. PLoS ONE 5:e12583 10.1371/journal.pone.0012583 20838446 PMC2933240

[B46] Moisander P. H. Beinart R. A. Hewson I. White A. E. Johnson K. S. Carlson C. A. (2010). Unicellular cyanobacterial distributions broaden the oceanic N_2_ fixation domain. Science 327, 1512–1514 10.1126/science.1185468 20185682

[B47] Montoya J. P. Voss M. Kahler P. Capone D. G. (1996). A simple, high-precision, high-sensitivity tracer assay for N_2_ fixation. Appl. Environ. Microbiol. 62, 986–993 16535283 10.1128/aem.62.3.986-993.1996 PMC1388808

[B48] Mulholland M. R. Bernhardt P. W. (2005). The effect of growth rate, phosphorus concentration, and temperature on N_2_ fixation, carbon fixation, and nitrogen release in continuous cultures of *Trichodesmium* IMS101. Limnol. Oceanogr. 50, 839–849 10.4319/lo.2005.50.3.0839

[B49] Mulholland M. R. Bernhardt P. W. Blanco-Garcia J. L. Mannino A. Hyde K. Mondragon E. (2012). Rates of dinitrogen fixation and the abundance of diazotrophs in North American coastal waters between Cape Hatteras and Georges Bank. Limnol. Oceanogr. 57, 1067–1083 10.4319/lo.2012.57.4.1067

[B50] Mulholland M. R. Bernhardt P. W. Heil C. A. Bronk D. A. O'Neil J. M. (2006). Nitrogen fixation and release of fixed nitrogen by Trichodesmium spp in the Gulf of Mexico. Limnol. Oceanogr. 51, 1762–1776 10.4319/lo.2006.51.4.1762

[B51] Mulholland M. R. Capone D. G. (1999). Nitrogen fixation, uptake and metabolism in natural and cultured populations of *Trichodesmium* spp. Mar. Ecol. Prog. Ser. 188, 33–49 10.3354/meps188033

[B52] Mulholland M. R. Ohki K. Capone D. G. (2001). Nutrient controls on nitrogen uptake and metabolism by natural populations and cultures of *Trichodesmium* (Cyanobacteria). J. Phycol. 37, 1001–1009 10.1046/j.1529-8817.2001.00080.x

[B53] O'Neil J. M. Capone D. G. (2008). “Nitrogen Cycling in Coral Reef Environments,” in Nitrogen in the Marine Environment, 2nd Edn., eds Capone D. Bronk D. Mulholland M. Carpenter E. (Amsterdam: Elsevier), 949–989

[B54] Orcutt K. M. Lipschultz F. Gundersen K. Arimoto R. Michaels A. F. Knap A. H. (2001). A seasonal study of the significance of N_2_ fixation by *Trichodesmium* spp. at the Bermuda Atlantic Time-series Study (BATS) site. Deep Sea Res. I. 48, 1583–1608

[B55] Paerl H. Prufert L. E. (1987). Oxygen-poor microzones as potential sites of microbial N_2_ fixation in nitrogen-depleted aerobic marine waters. Appl. Environ. Microbiol. 53, 1078–1087 16347337 10.1128/aem.53.5.1078-1087.1987 PMC203813

[B56] Passow U. (2002). Production of transparent exopolymer particles (TEP) by phyto- and bacterioplankton. Mar. Ecol. Prog. Ser. 236, 1–12 10.3354/meps236001

[B57] Passow U. Alldredge A. L. (1995). A dye-binding assay for the spectrophotometric measurement of transparent exopolymer particles (TEP). Limnol. Oceanogr. 40, 1326–1335 10.4319/lo.1995.40.7.1326

[B58] Paytan A. Mackey K. R. M. Chen Y. Lima I. D. Doney S. C. Mahowald N. (2009). Toxicity of atmospheric aerosols on marine phytoplankton. Proc. Natl. Acad. Sci. U.S.A 106, 4601–4605 10.1073/pnas.0811486106 19273845 PMC2653564

[B59] Post A. F. Dedej Z. Gottlieb R. Li H. Thomas D. N. El-Absawi M. (2002). Spatial and temporal distribution of *Trichodesmium* spp in the stratified Gulf of Aqaba, Red Sea. Mar. Ecol. Prog. Ser. 239, 241–250 10.3354/meps239241

[B60] Postgate J. (1998). “Nitrogen fixation,” in Nitrogen Fixation, 3rd Edn., ed Postgate J. (London: Cambridge University Press), 124.

[B61] Rahav E. Herut B. Stambler N. Bar-Zeev E. Mulholland M. R. Berman-Frank I. (2013a). Uncoupling between dinitrogen fixation and primary productivity in the Eastern Mediterranean Sea. J. Geophys. Res. 118, 1–8 10.1002/jgrg.20023

[B62] Rahav E. Herut B. Levi A. Mulholland M. R. Berman-Frank I. (2013b). Springtime contribution of dinitrogen fixation to primary production across the Mediterranean Sea. Ocean Sci. 9, 1–10 10.5194/os-9-489-2013

[B63] Rees A. P. Gilbert J. A. Kelly-Gerreyn B. A. (2009). Nitrogen fixation in the western English Channel (NE Atlantic Ocean). Mar. Ecol. Prog. Ser. 374, 7–12 10.3354/meps07771

[B64] Riemann L. Farnelid H. Steward G. F. (2010). Nitrogenase genes in non-cyanobacterial plankton: prevalence, diversity and regulation in marine waters. Aquat. Microb. Ecol. 61, 225–237 10.3354/ame01431

[B65] Simon M. Alldredge A. L. Azam F. (1990). Bacterial carbon dynamics on marine snow. Mar. Ecol. Prog. Ser. 65, 205–211 10.3354/meps065205

[B66] Tamura K. Dudley J. Nei M. Kumar S. (2007). MEGA4: molecular evolutionary genetics analysis (MEGA) software version 4.0. Mol. Biol. Evol. 24, 1596–1599 10.1093/molbev/msm092 17488738

[B67] Tanaka T. Thingstad T. F. Christaki U. Colombet J. Cornet-Barthaux V. Courties C. (2011). Lack of P-limitation of phytoplankton and heterotrophic prokaryotes in surface waters of three anticyclonic eddies in the stratified Mediterranean Sea. Biogeosciences 8, 525–538 10.5194/bg-8-525-2011

[B68] Voss M. Croot P. Lochte K. Mills M. Peeken I. (2004). Patterns of nitrogen fixation along 10N in the tropical Atlantic. Geophys. Res. Lett. 31 10.1029/2004gl020127

[B69] Ward B. B. Devol A. H. Rich J. J. Chang B. X. Bulow S. E. Naik H. (2009). Denitrification as the dominant nitrogen loss process in the Arabian Sea. Nature 461, 78–81 10.1038/nature08276 19727197

[B70] Weiss R. F. (1970). Solubility of nitrogen, oxygen and argon in water and seawater. Deep-Sea Res. 17, 721–735

[B71] Wilson S. T. Boettjer D. Church M. J. Karl D. M. (2012). Comparative assessment of nitrogen fixation methodologies, conducted in the oligotrophic North Pacific Ocean. Appl. Environ. Microbiol. 78, 6516–6523 10.1128/AEM.01146-12 22773638 PMC3426697

[B72] Wu J. F. Chung S.-W. Wen L.-S. Liu K.-K. Chen Y.-L. L. Chen H.-Y. (2003). Dissolved inorganic phosphorus, dissolved iron, and *Trichodesmium* in the oligotrophic South China Sea. Global Biogeochem. Cycles 17:1008 10.1029/2002GB001924

[B73] Yogev T. Rahav E. Bar-Zeev E. Man-Aharonovich D. Stambler N. Kress N. (2011). Is dinitrogen fixation significant in the Levantine Basin, East Mediterranean Sea. Environ. Microbiol. 13, 854–871 10.1111/j.1462-2920.2010.02402.x 21244595

[B74] Zehr J. P. Bench S. R. Carter B. J. Hewson I. Niazi F. Shi T. (2008). Globally distributed uncultivated oceanic N2-fixing cyanobacteria lack oxygenic Photosystem, II. Science 322, 1110–1112 10.1126/science.1165340 19008448

[B75] Zehr J. P. Jenkins B. D. Short S. M. Steward G. F. (2003). Nitrogenase gene diversity and microbial community structure: a cross-system comparison. Environ. Microbiol. 5, 539–554 10.1046/j.1462-2920.2003.00451.x 12823187

[B76] Zehr J. P. McReynolds L. A. (1989). Use of degenerate oligonucleotides for amplification of the *nifH* gene from the marine cyanobacterium *Trichodesmium-thiebautii*. Appl. Environ. Microbiol. 55, 2522–2526 2513774 10.1128/aem.55.10.2522-2526.1989 PMC203115

[B77] Zehr J. P. Ward B. B. (2002). Nitrogen cycling in the ocean: new perspectives on processes and paradigms. Appl. Environ. Microbiol. 68, 1015–1024 10.1128/AEM.68.3.1015-1024.2002 11872445 PMC123768

